# Supplementary figures and images for: Mapping the single-cell landscape of acral melanoma and analysis of the molecular regulatory network of the tumor microenvironments
Source: eLife. 2022 Jul 27;11:e78616. doi: 10.7554/eLife.78616 (PMC9398445; doi:10.7554/eLife.78616)

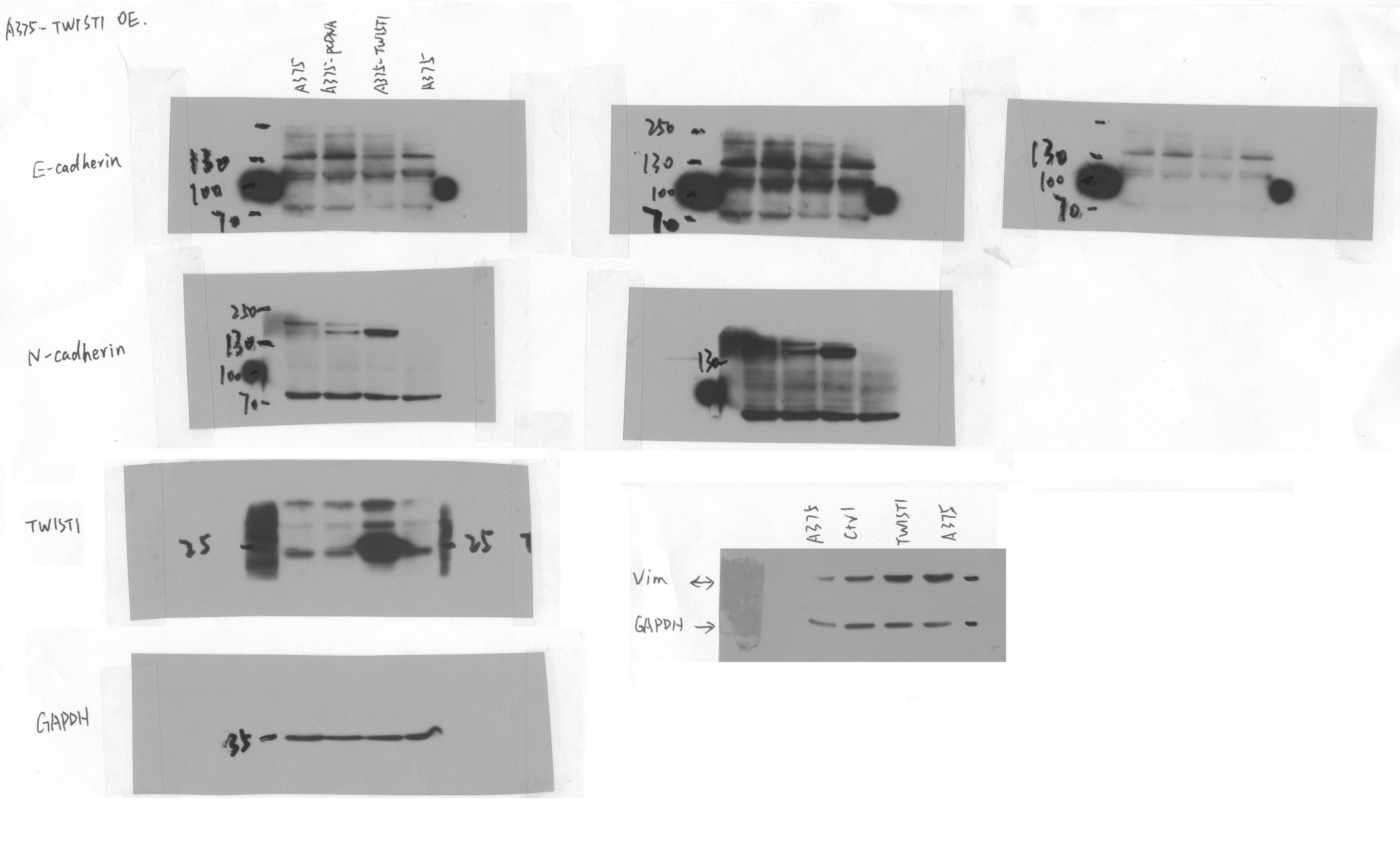

Supplement: Figure 3—figure supplement 1—source data 1. [file elife-78616-fig3-figsupp1-data1.zip › Figure 3-figure supplement 1-source data/TWIST/Figure 3-figure supplement 1-source data 1 EMT WB.tif]

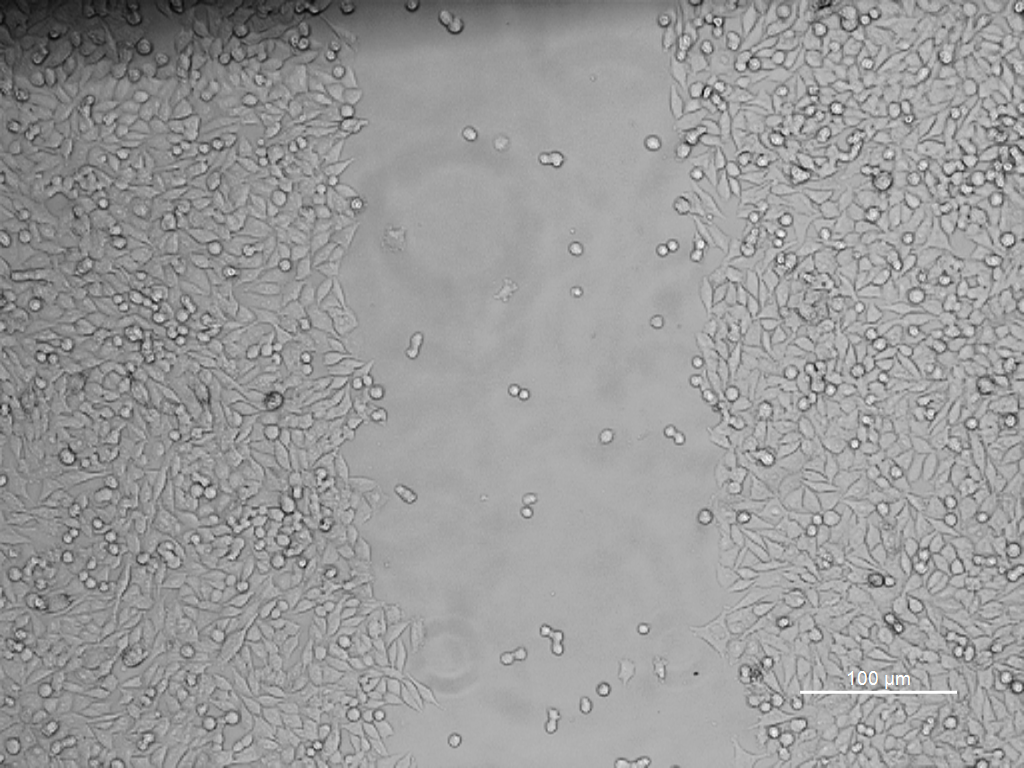

Supplement: Figure 3—figure supplement 1—source data 1. [file elife-78616-fig3-figsupp1-data1.zip › Figure 3-figure supplement 1-source data/TWIST/Figure 3-figure supplement 1-source data 2 woundhealing assay/A375 pcDNA 2-0003 48H.tif]

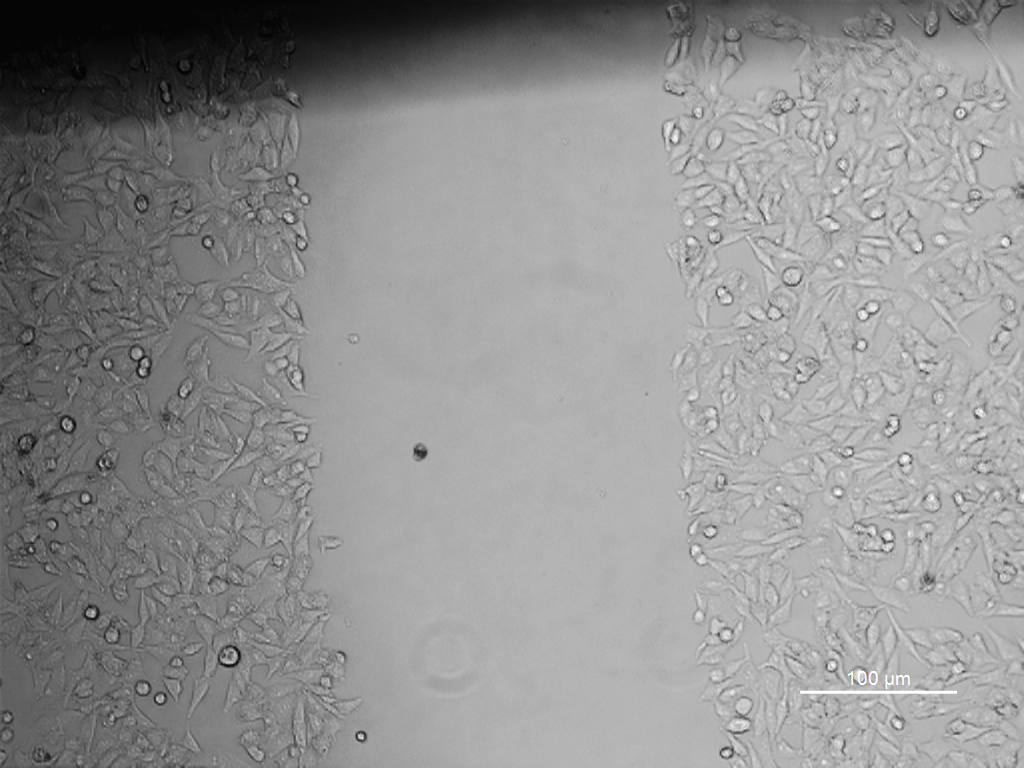

Supplement: Figure 3—figure supplement 1—source data 1. [file elife-78616-fig3-figsupp1-data1.zip › Figure 3-figure supplement 1-source data/TWIST/Figure 3-figure supplement 1-source data 2 woundhealing assay/A375 pcDNA 2-0003.tif]

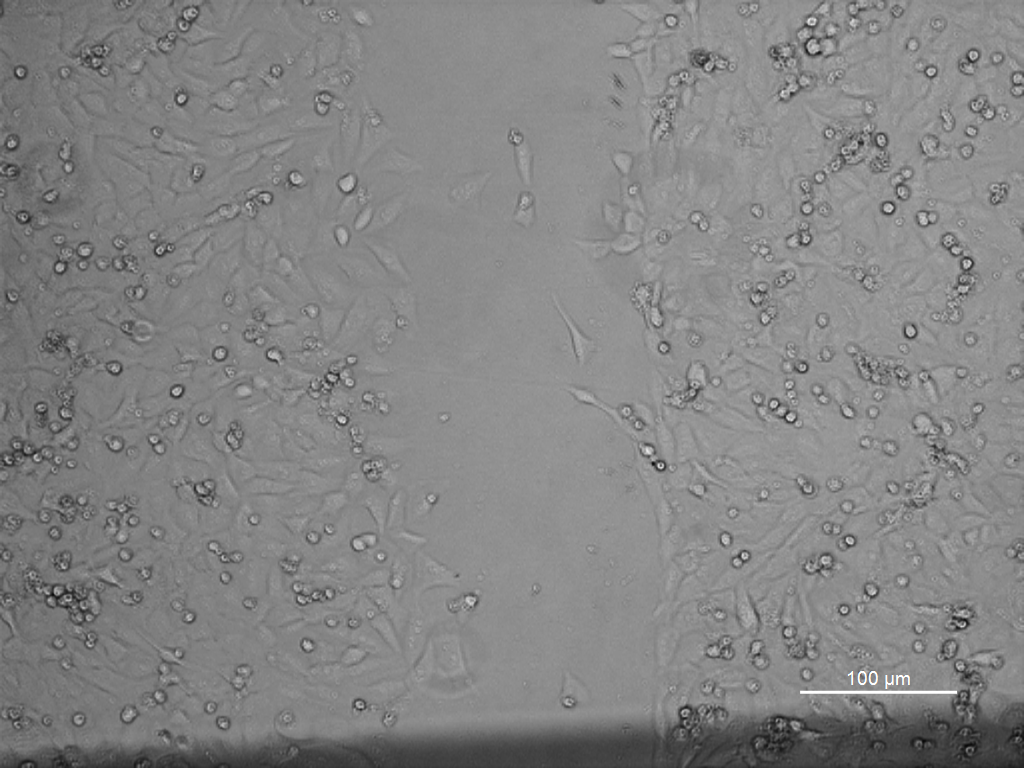

Supplement: Figure 3—figure supplement 1—source data 1. [file elife-78616-fig3-figsupp1-data1.zip › Figure 3-figure supplement 1-source data/TWIST/Figure 3-figure supplement 1-source data 2 woundhealing assay/A375 pcDNA-TWIST1 1-0006 48H.tif]

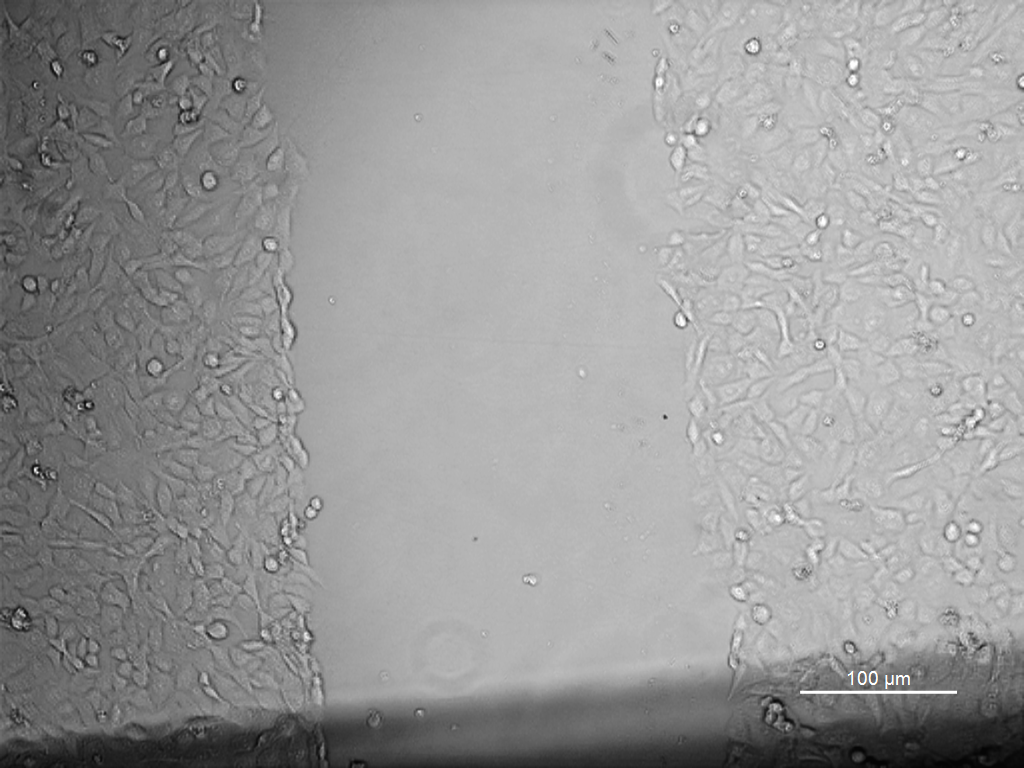

Supplement: Figure 3—figure supplement 1—source data 1. [file elife-78616-fig3-figsupp1-data1.zip › Figure 3-figure supplement 1-source data/TWIST/Figure 3-figure supplement 1-source data 2 woundhealing assay/A375 pcDNA-TWIST1 1-0006.tif]

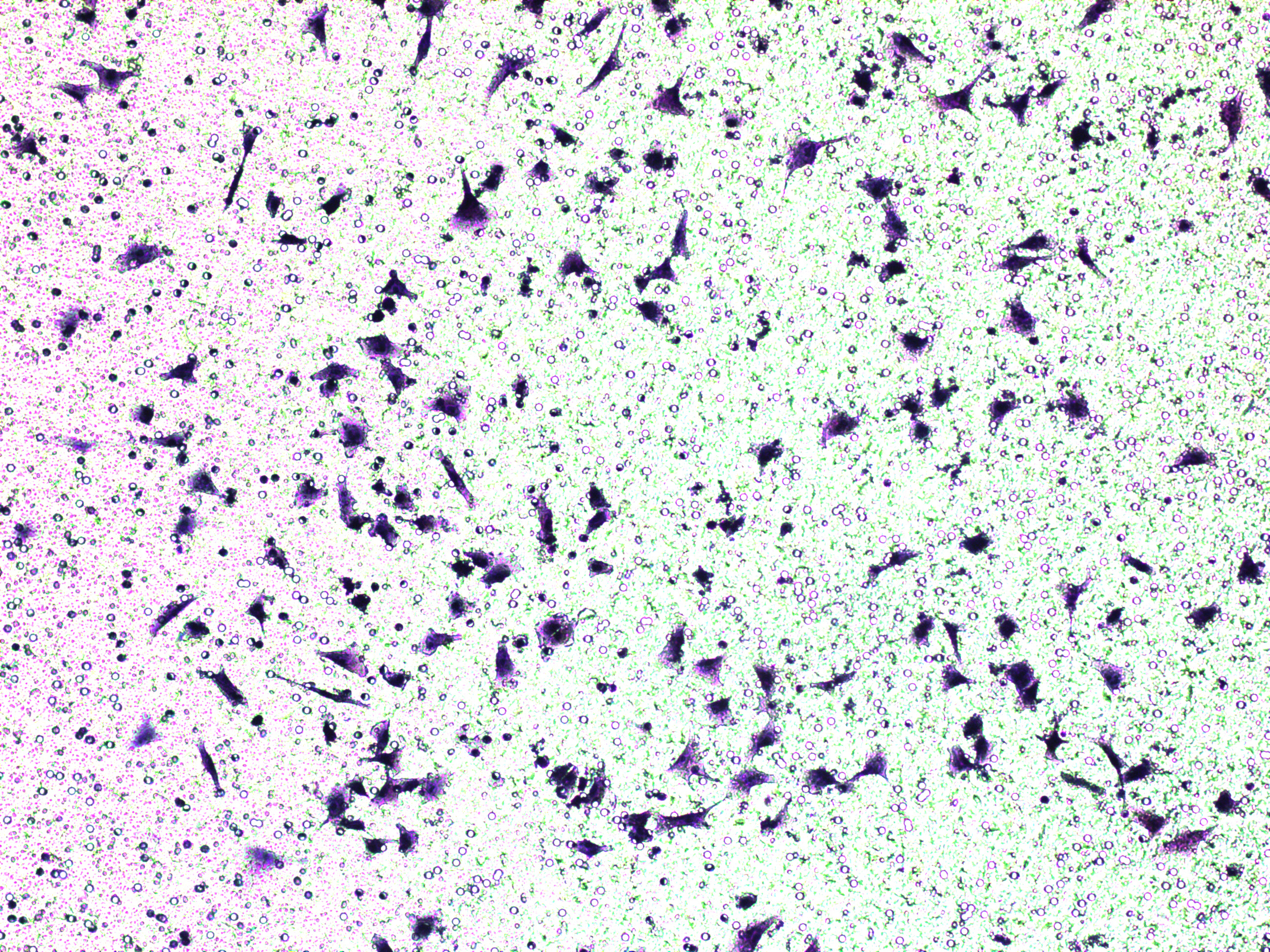

Supplement: Figure 3—figure supplement 1—source data 1. [file elife-78616-fig3-figsupp1-data1.zip › Figure 3-figure supplement 1-source data/TWIST/Figure 3-figure supplement 1-source data 3 Transwell/A375 TWIST1 migration/ctrl--2-10X-2-5.jpg]

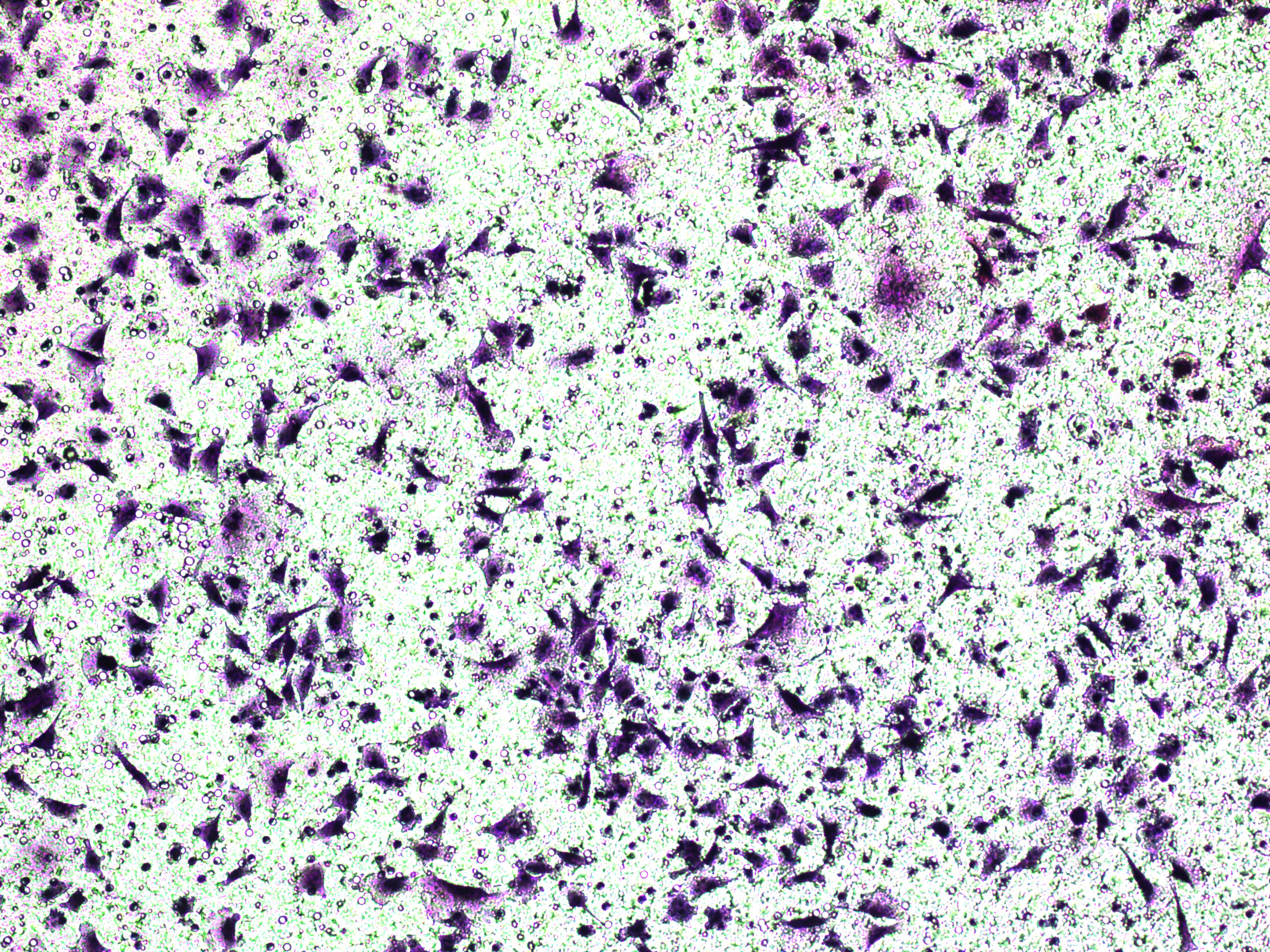

Supplement: Figure 3—figure supplement 1—source data 1. [file elife-78616-fig3-figsupp1-data1.zip › Figure 3-figure supplement 1-source data/TWIST/Figure 3-figure supplement 1-source data 3 Transwell/A375 TWIST1 migration/TWIST1--3-10X-6.jpg]

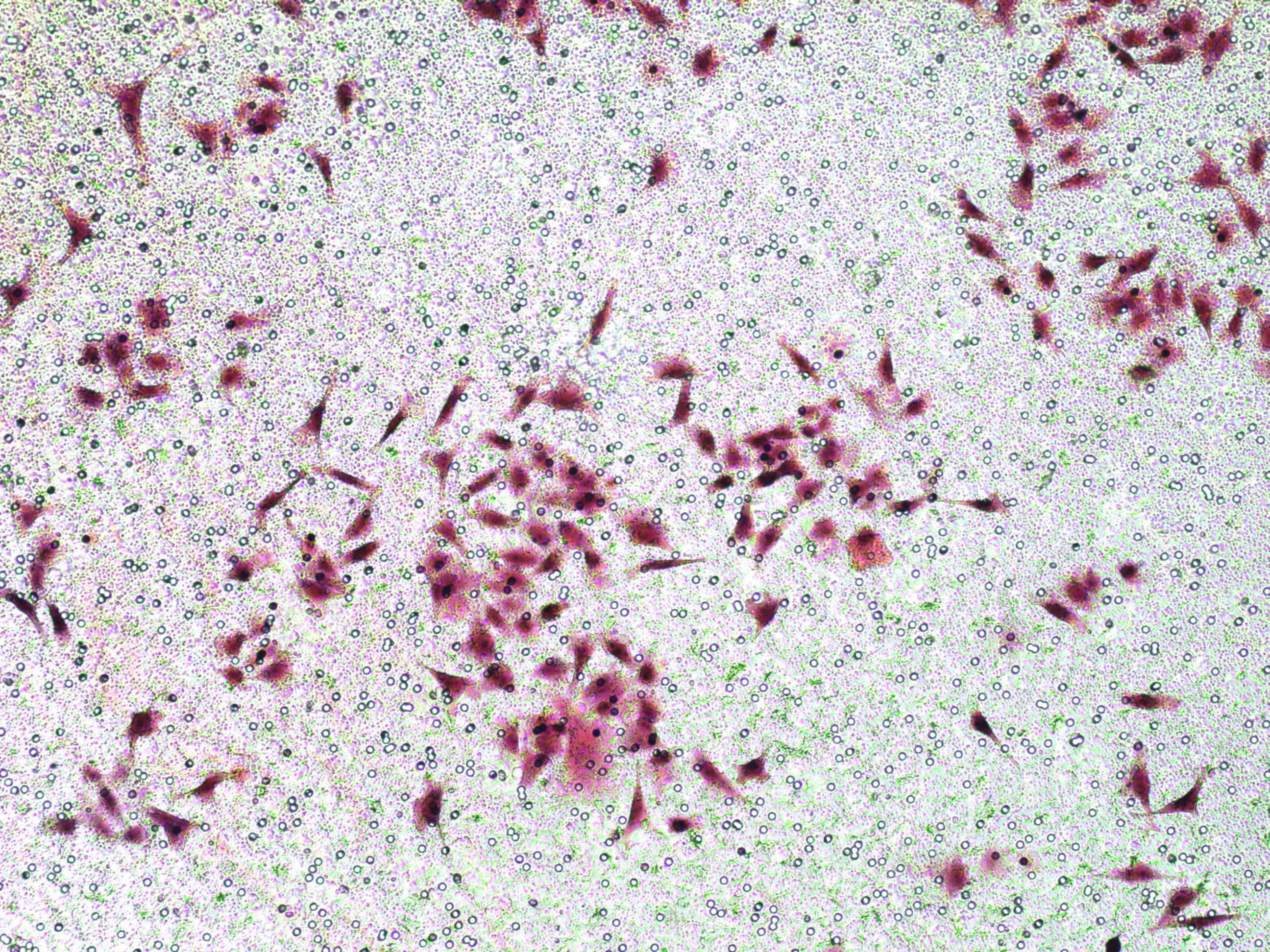

Supplement: Figure 3—figure supplement 1—source data 1. [file elife-78616-fig3-figsupp1-data1.zip › Figure 3-figure supplement 1-source data/TWIST/Figure 3-figure supplement 1-source data 3 Transwell/A375-TWIST1 invasion/A375-ctrl-invasion.tif]

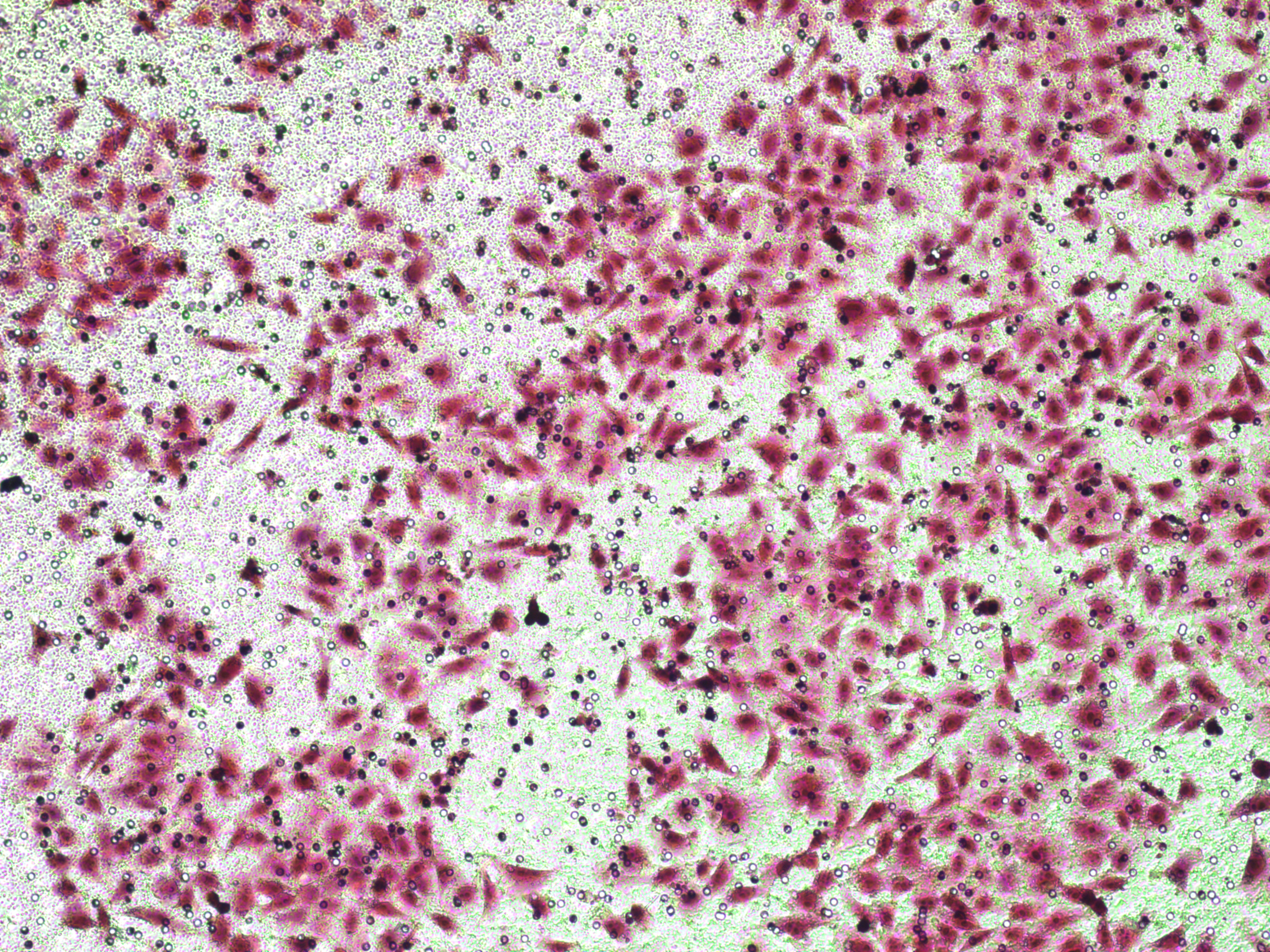

Supplement: Figure 3—figure supplement 1—source data 1. [file elife-78616-fig3-figsupp1-data1.zip › Figure 3-figure supplement 1-source data/TWIST/Figure 3-figure supplement 1-source data 3 Transwell/A375-TWIST1 invasion/A375-TWIST1-invasion.tif]

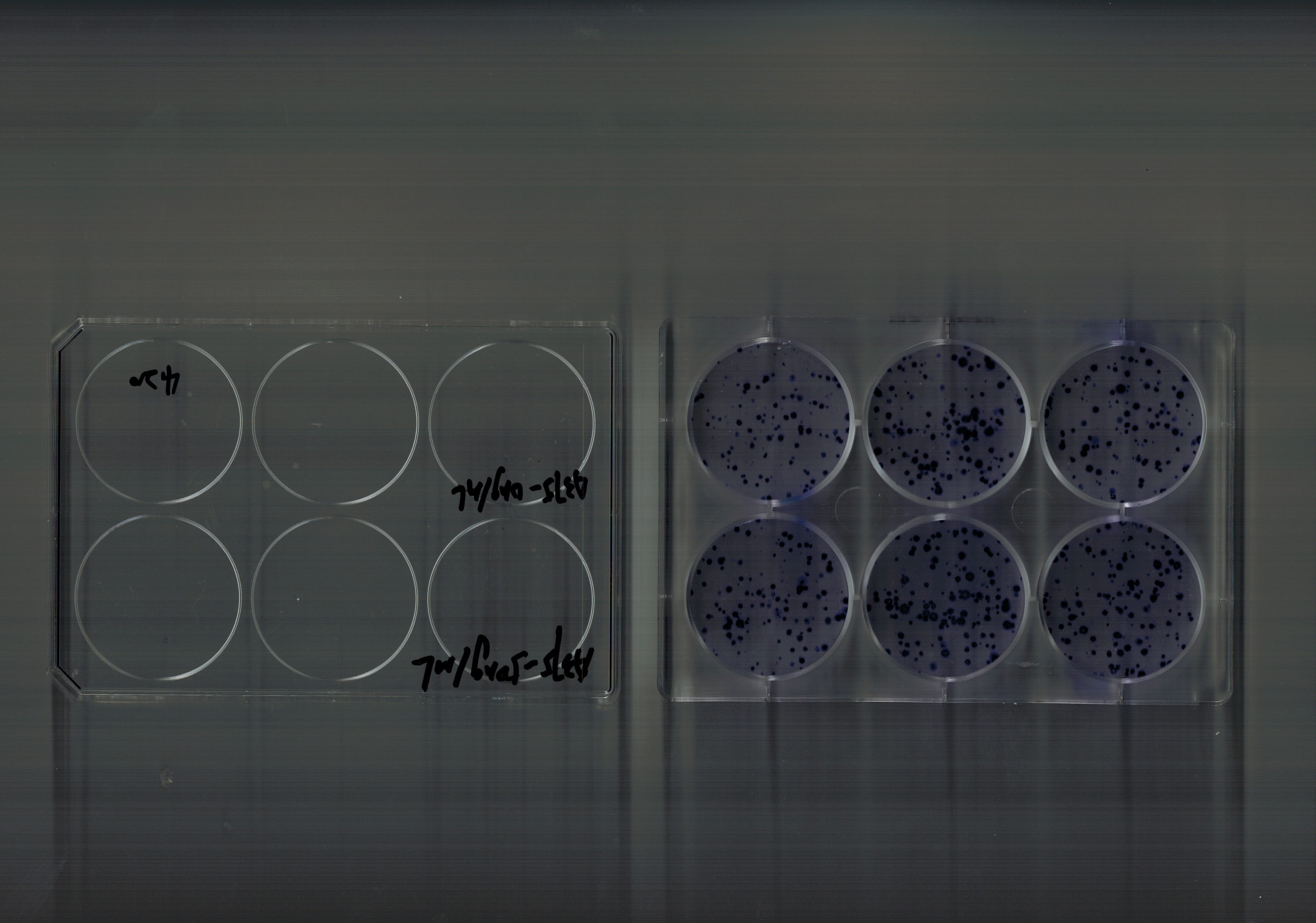

Supplement: Figure 7—source data 1. [file elife-78616-fig7-data1.zip › Figure 7-source data 1/Clone formation assay/formal/4.20.jpg]

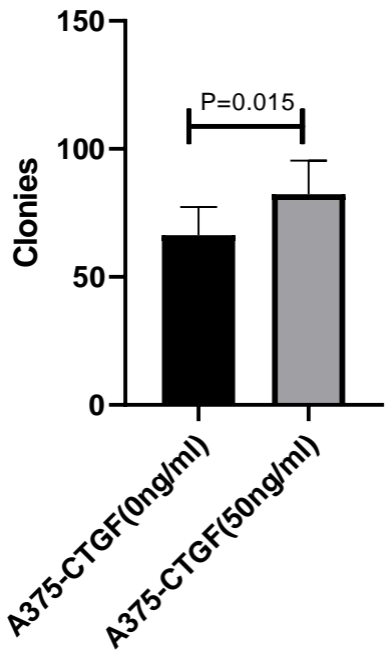

Supplement: Figure 7—source data 1. [file elife-78616-fig7-data1.zip › Figure 7-source data 1/Clone formation assay/formal/4.20results.pdf]

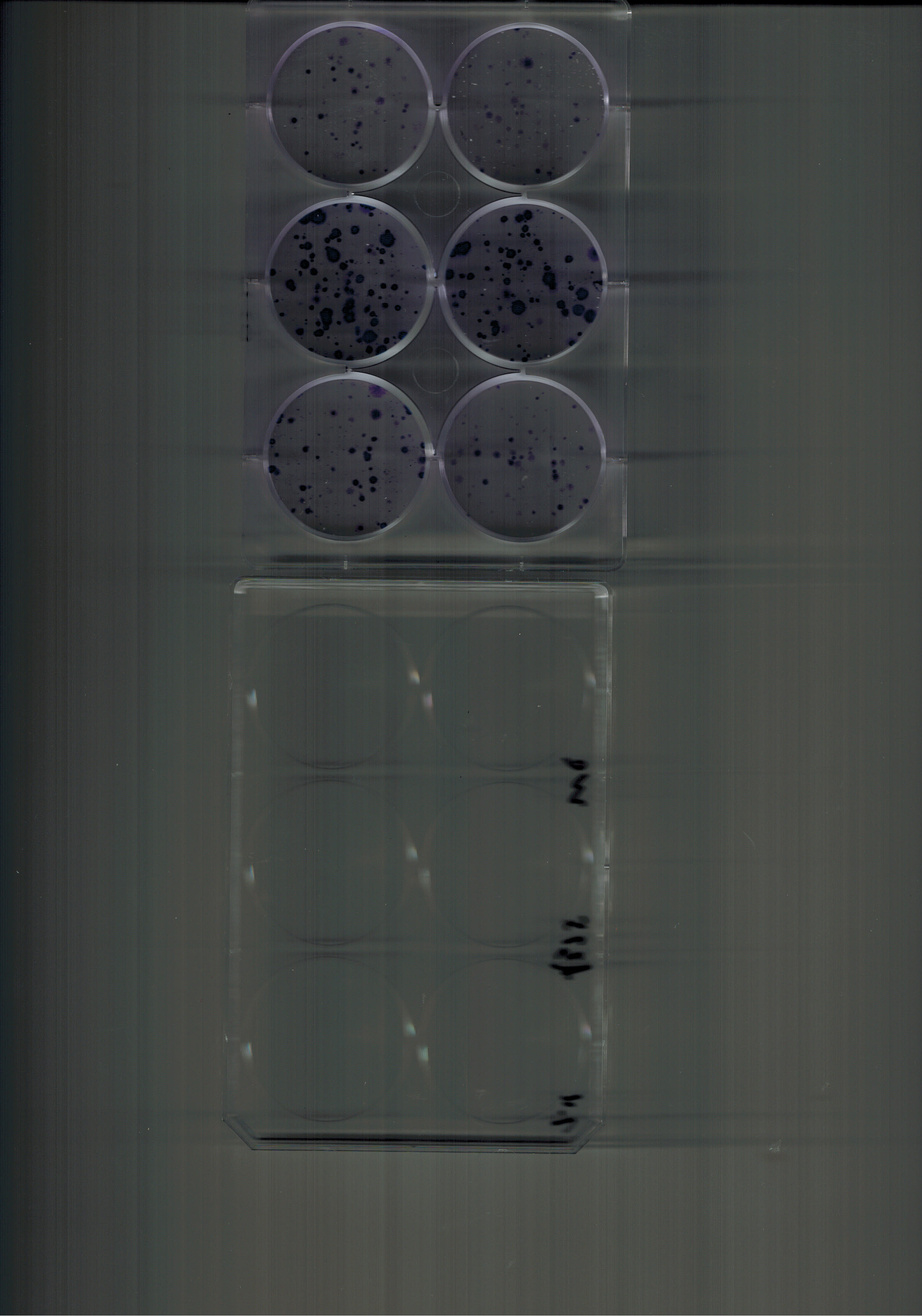

Supplement: Figure 7—source data 1. [file elife-78616-fig7-data1.zip › Figure 7-source data 1/Clone formation assay/repetition/7.11-1/A375.jpg]

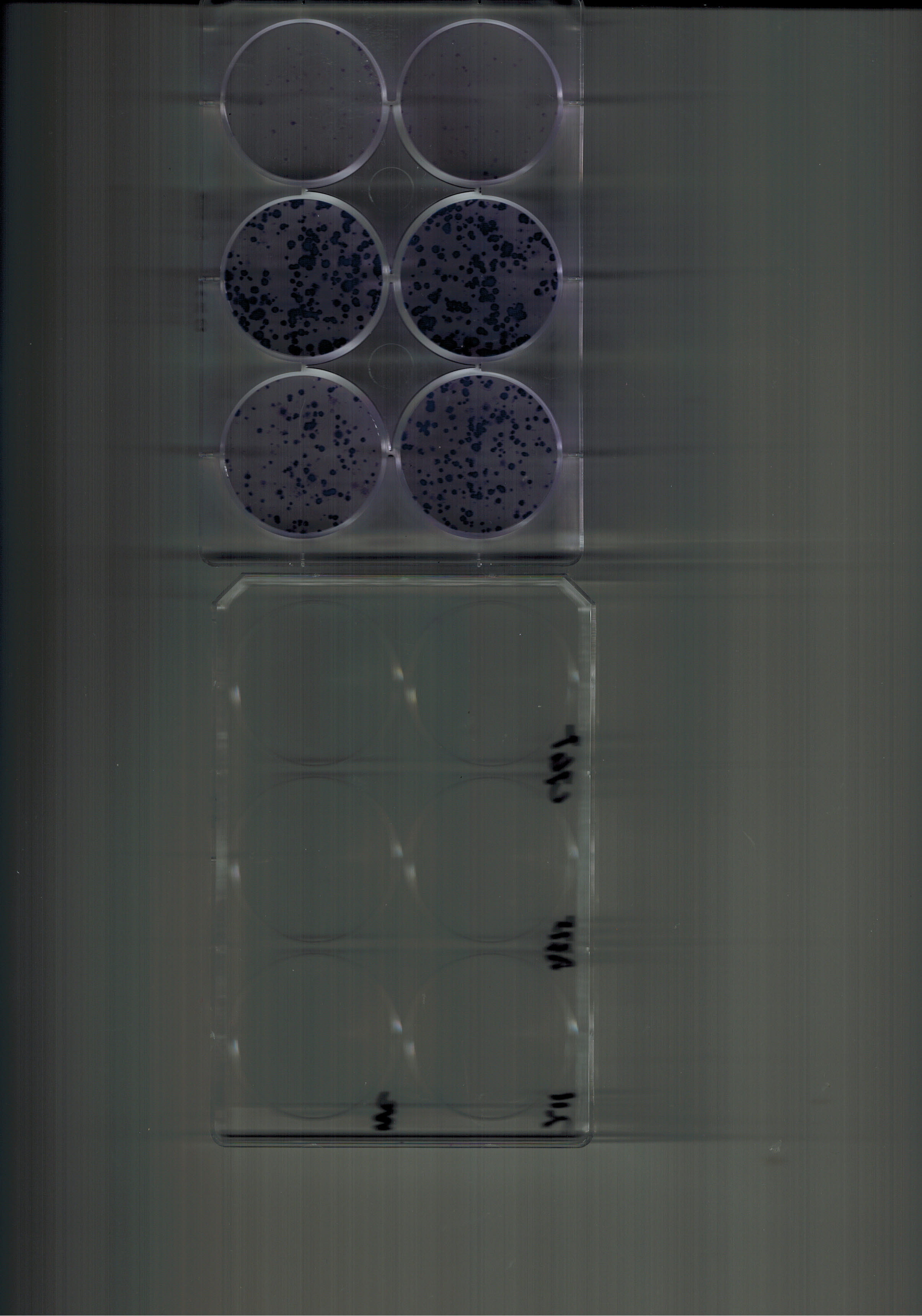

Supplement: Figure 7—source data 1. [file elife-78616-fig7-data1.zip › Figure 7-source data 1/Clone formation assay/repetition/7.11-1/CTGF-50.jpg]

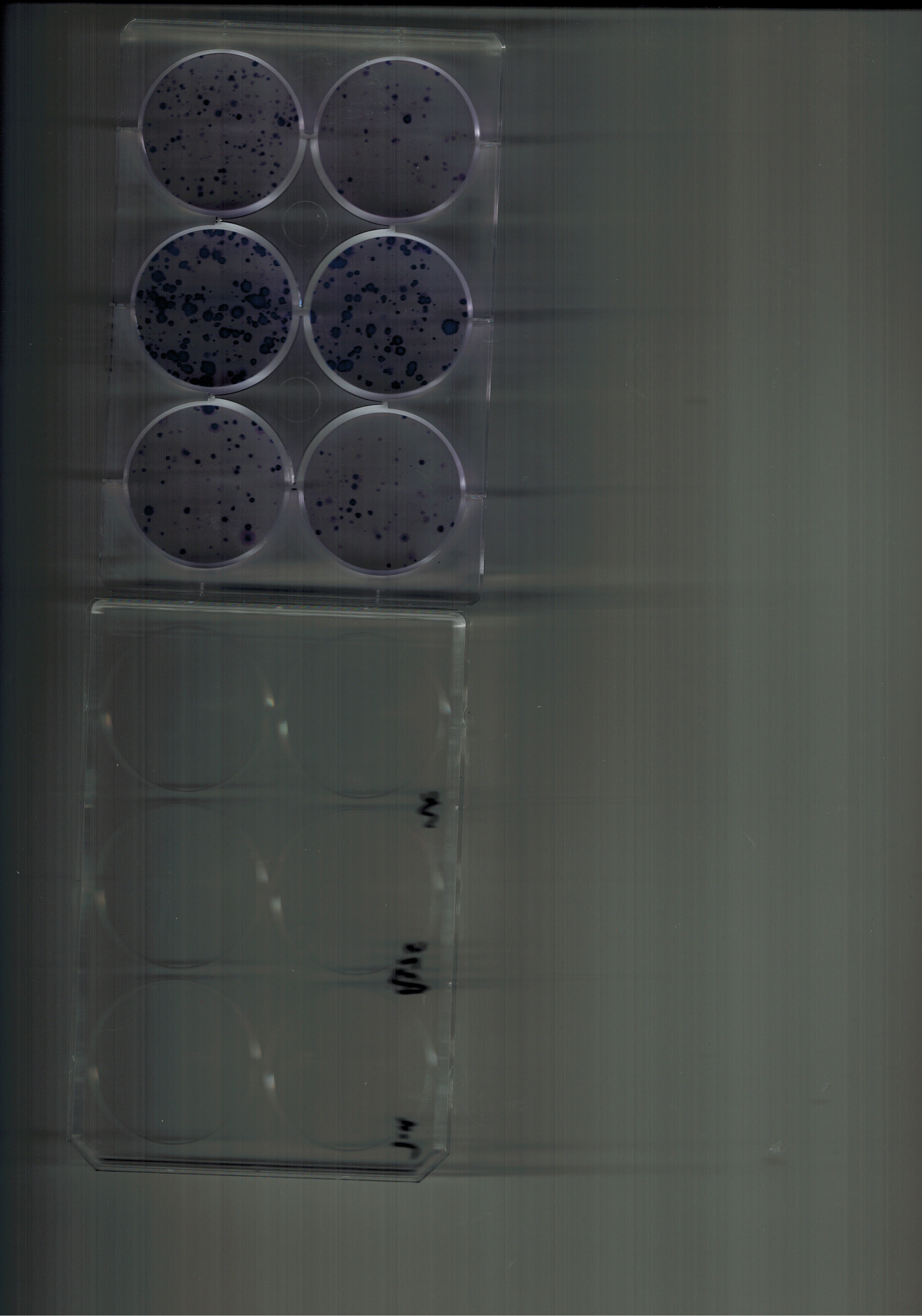

Supplement: Figure 7—source data 1. [file elife-78616-fig7-data1.zip › Figure 7-source data 1/Clone formation assay/repetition/7.11-2/A375 (2).jpg]

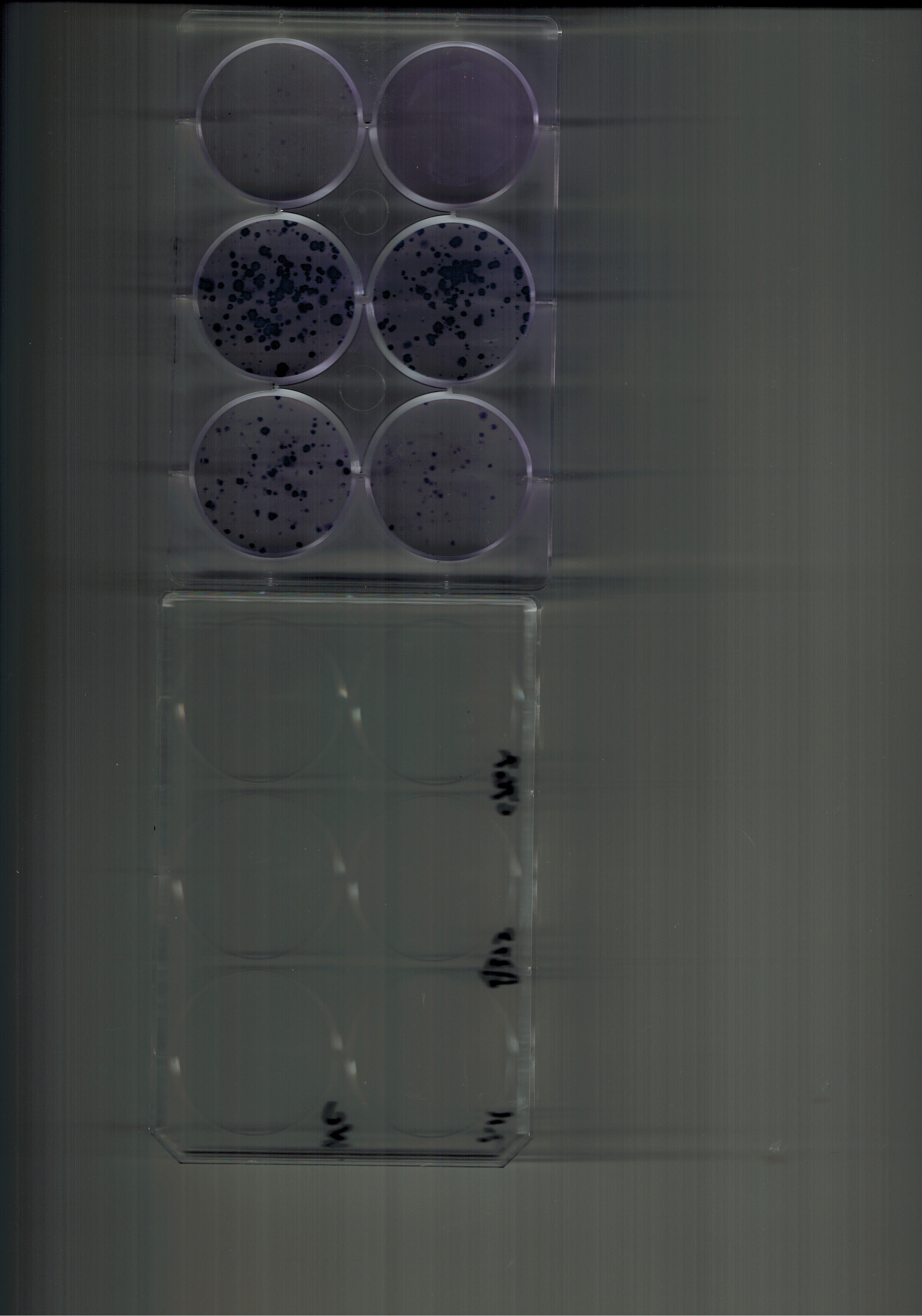

Supplement: Figure 7—source data 1. [file elife-78616-fig7-data1.zip › Figure 7-source data 1/Clone formation assay/repetition/7.11-2/CTGF-50 (2).jpg]

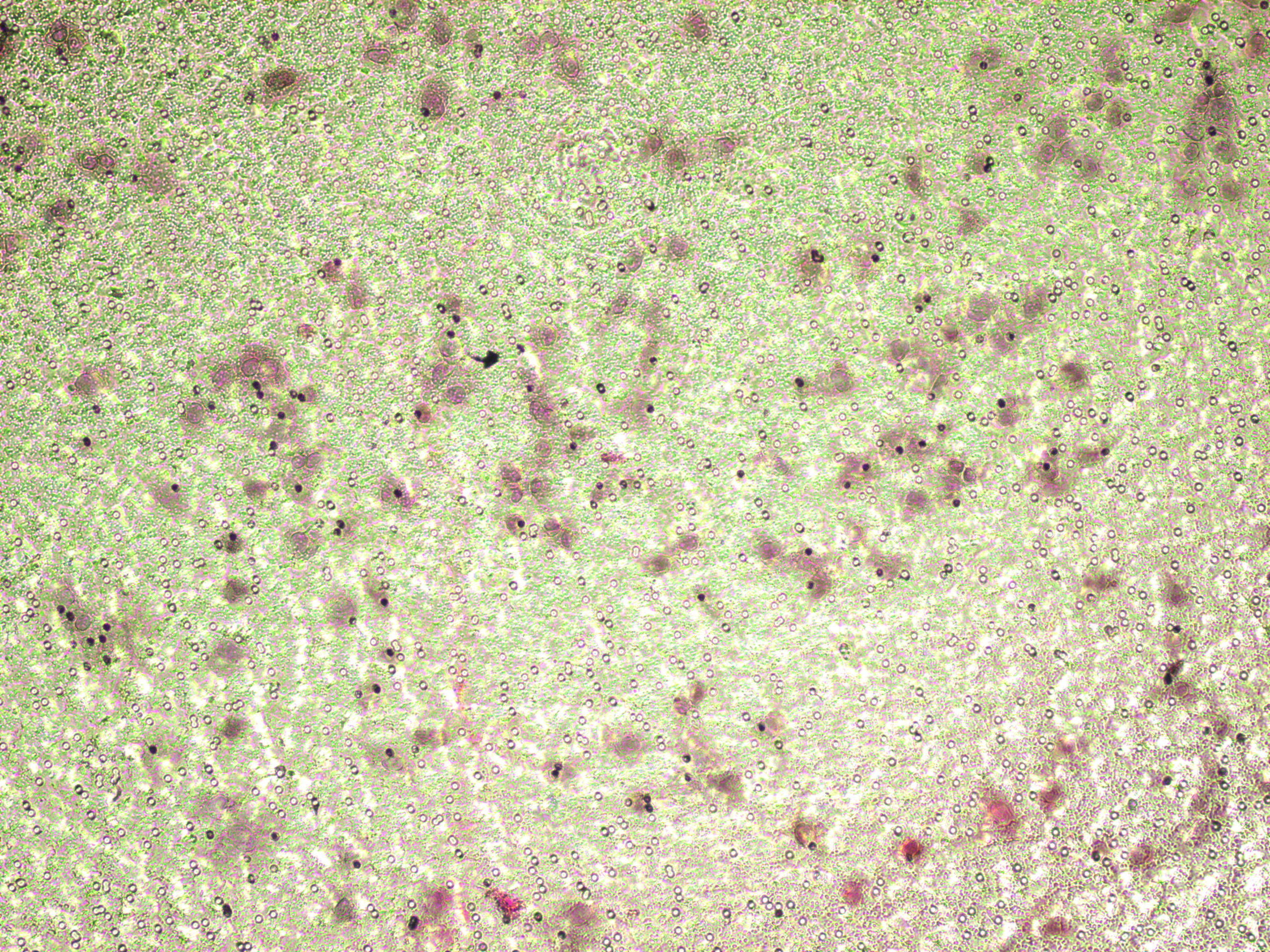

Supplement: Figure 7—source data 2. [file elife-78616-fig7-data2.zip › Figure 7-source data 2/Transwell/10X A3-0ng per ml.tif]

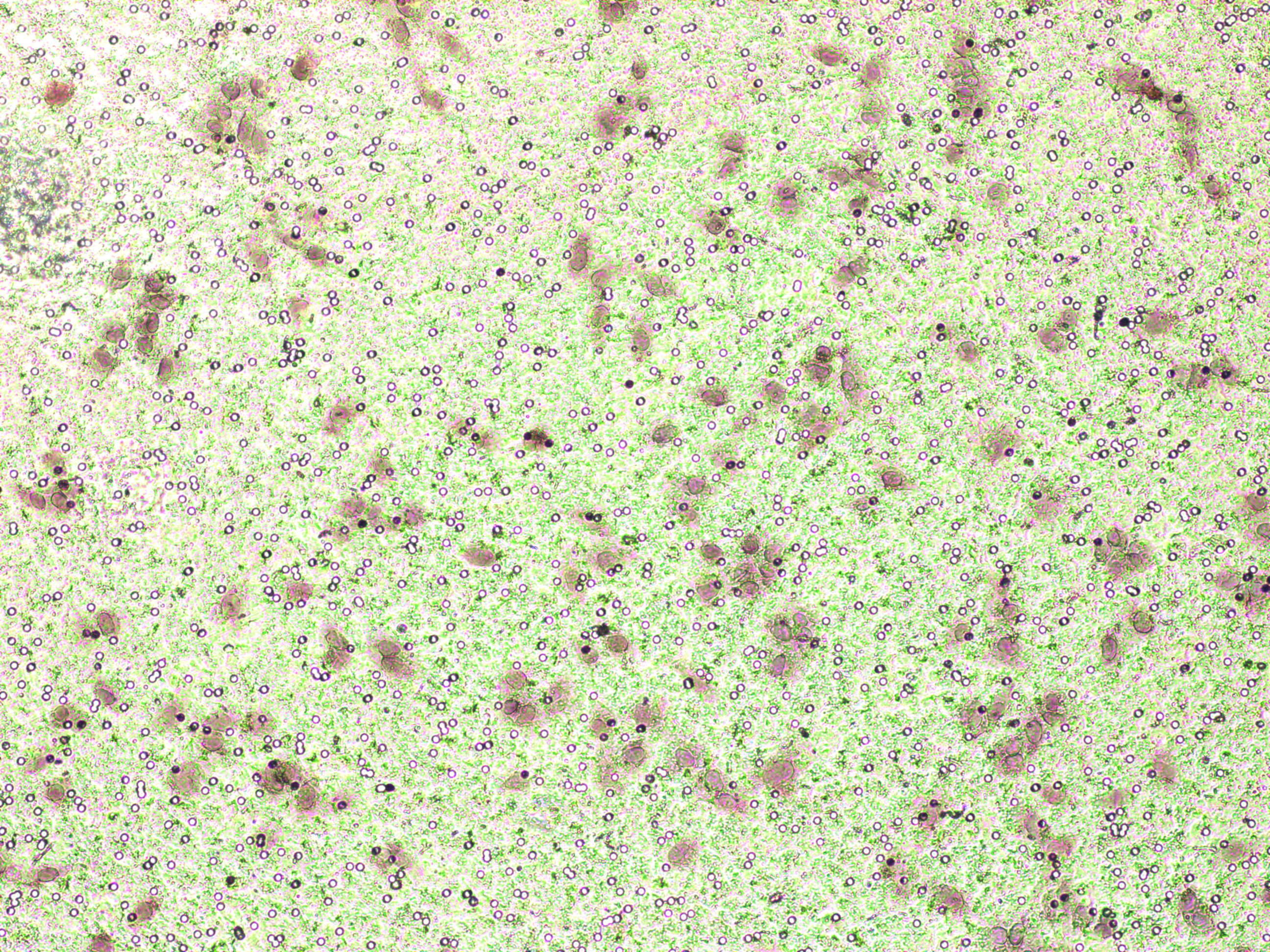

Supplement: Figure 7—source data 2. [file elife-78616-fig7-data2.zip › Figure 7-source data 2/Transwell/10X A3-50ng per ml.tif]

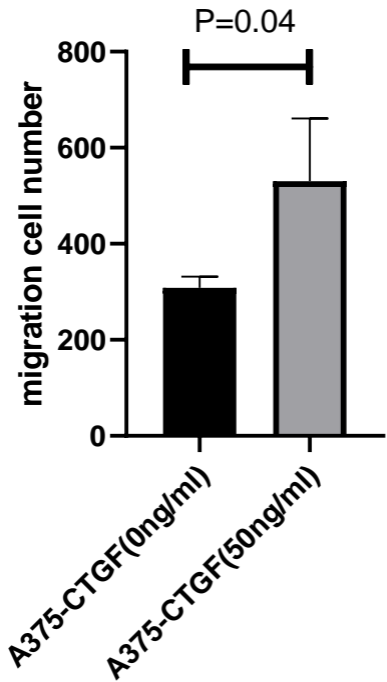

Supplement: Figure 7—source data 2. [file elife-78616-fig7-data2.zip › Figure 7-source data 2/Transwell/num.pdf]
